# Biomechanical Evaluation and Strength Test of 3D-Printed Foot Orthoses

**DOI:** 10.1155/2019/4989534

**Published:** 2019-12-07

**Authors:** Kuang-Wei Lin, Chia-Jung Hu, Wen-Wen Yang, Li-Wei Chou, Shun-Hwa Wei, Chen-Sheng Chen, Pi-Chang Sun

**Affiliations:** ^1^Department of Physical Therapy and Assistive Technology, National Yang-Ming University, Taipei, Taiwan; ^2^Department of Sports Medicine, China Medical University, Taichung, Taiwan; ^3^Department of Rehabilitation Medicine, Taipei City Hospital, Taipei, Taiwan; ^4^Faculty of Medicine, National Yang-Ming University, Taipei, Taiwan

## Abstract

Foot orthoses (FOs) are commonly used as interventions for individuals with flatfoot. Advances in technologies such as three-dimensional (3D) scanning and 3D printing have facilitated the fabrication of custom FOs. However, few studies have been conducted on the mechanical properties and biomechanical effects of 3D-printed FOs. The purposes of this study were to evaluate the mechanical properties of 3D-printed FOs and determine their biomechanical effects in individuals with flexible flatfoot. During mechanical testing, a total of 18 FO samples with three orientations (0°, 45°, and 90°) were fabricated and tested. The maximum compressive load and stiffness were calculated. During a motion capture experiment, 12 individuals with flatfoot were enrolled, and the 3D-printed FOs were used as interventions. Kinematic and kinetic data were collected during walking by using an optical motion capture system. A one-way analysis of variance was performed to compare the mechanical parameters among the three build orientations. A paired *t*-test was conducted to compare the biomechanical variables under two conditions: walking in standard shoes (Shoe) and walking in shoes embedded with FOs (Shoe+FO). The results indicated that the 45° build orientation produced the strongest FOs. In addition, the maximum ankle evertor and external rotator moments under the Shoe+FO condition were significantly reduced by 35% and 16%, respectively, but the maximum ankle plantar flexor moments increased by 3%, compared with the Shoe condition. No significant difference in ground reaction force was observed between the two conditions. This study demonstrated that 3D-printed FOs could alter the ankle joint moments during gait.

## 1. Introduction

Foot orthoses (FOs) are commonly used as interventions for individuals with flexible flatfoot [1, 2]. Wearing FOs might improve the pain scores [3] and alter the kinematics and kinetics of the rearfoot; for example, FOs reduce the peak rearfoot eversion [3, 4] and joint moment in the frontal plane [5]. However, a study indicated that the same shoe insert interventions produce substantially different effects for dissimilar individuals [6]. The actual prescription of orthotic devices is patient specific; this is because various levels of malalignments likely require different FO designs. According to the current manufacturing method, FOs can be either custom fabricated or prefabricated. Although prefabricated FOs are less expensive and are readily available as off-the-shelf products, custom FOs normally exhibit a better fit to an individual's foot and are more effective than prefabricated FOs [7].

Custom FOs can be prescribed in three types: soft, semirigid, and rigid. The traditional plaster-molding and vacuum-forming processes are used for fabricating rigid or semirigid FOs. For fabricating a conventional rigid FO, the foot is pressed into a foam box to create a negative impression of the plantar surface. The negative impression is used as a mold for plaster to produce the positive foot model. The positive model is draped with heated shell material and molded around the model using a vacuum press. The extra material around the edges is trimmed to complete the FO. Therefore, the manufacturing process of custom FOs is complicated, highly laborious, and time consuming.

Contrary to the traditional manufacturing of custom FOs, the technology of three-dimensional (3D) printing combined with 3D scanning is deal for mass customization, and this is because it provides the potential for fabricating customized FOs at relatively low prices and eliminates much of the labor [7]. Advances in technologies such as 3D scanning, computer-aided design, and 3D printing have enabled the fabrication of custom FOs [8, 9]. The FOs need sufficient mechanical strength to bear the body weight during walking. Ethylene vinyl acetate (EVA) or polypropylene (PP) is most often used to fabricate traditional FOs. The materials of 3D-printed FOs are different from the conventional FOs, and polylactic acid (PLA) is one of the most popular materials used in desktop 3D printing. Moreover, according to our review of the literature, different raster orientation angles could affect the mechanical properties of 3D-printed parts [10, 11]. Therefore, the mechanical properties of FOs fabricated under specific build orientations should be further tested to confirm its strength.

Although 3D printing technology demonstrates potential in the fabrication of custom FOs, computer-aided equipment and software are inaccessible to most clinical staff members because of a lack of engineering skills and high cost of acquisition. Moreover, the biomechanical effects of 3D-printed FOs remain unclear. The performance of human walking when wearing 3D-printed FOs should be tested. Therefore, in order to fully understand the effects of 3D-printed FOs, we merged mechanical testing and human motion analysis into this study. The purposes of this study were to fabricate FOs using low-cost 3D printing techniques and evaluate the mechanical properties and biomechanical effects of the 3D-printed FOs in individuals with flexible flatfoot.

## 2. Materials and Methods

### 2.1. Fabrication of 3D-Printed FOs

**Figure 1:**
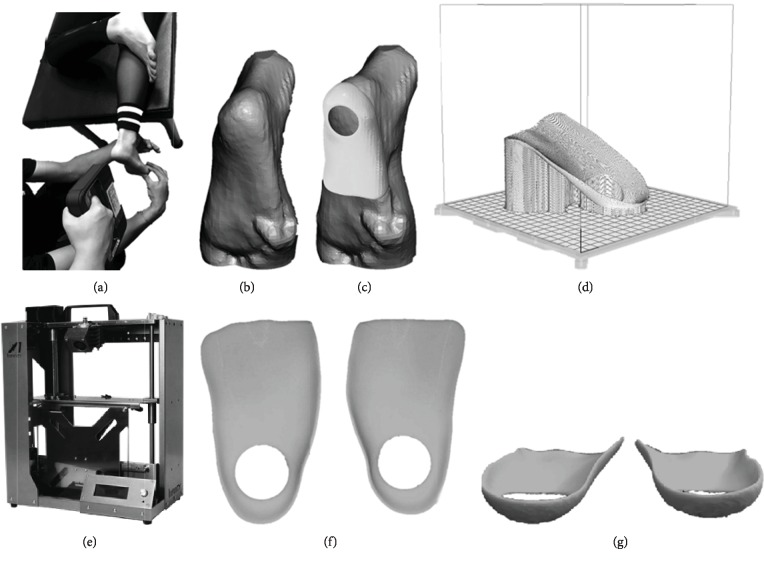
Fabrication of the 3D-printed FOs. (a) 3D scan of the foot in the subtalar neutral position. (b) Geometry of the foot exported as an STL file. (c) Extraction of the FO shape from the foot model. (d) Solid FO model imported into Cura software to be sliced and output as G-Code. (e) FO printed using an Infinity X1 3D printer. (f) Top view of the 3D-printed FO. (g) Rear view of the 3D-printed FO.

Figure 1 illustrates the procedure for fabricating FOs using a 3D printer. First, a participant's foot and ankle were maintained in the subtalar neutral position while a 3D scanner (SENSE, 3D System Inc., South Carolina, USA) scanned the foot; the scanning result was exported as a stereolithography (STL) file. The STL file was smoothed and edited using Meshmixer software (Autodesk, Inc., California, USA) before being sent to the 3D printer. The thickness of the FO model was set to 2 mm. The medial and lateral longitudinal arch of foot is covered by the FO. The FO model was printed in PLA filament, layered at 0.2 mm along with a shell thickness of 0.8 mm, fill density of 90%, and nozzle temperature of 200°C, by using a fused deposition modeling 3D printer (Infinity X1, INFINITY3DP, Kaohsiung, Taiwan). The build parameters of the 3D printer were defined using the open-source slicer program Ultimaker Cura 3.3 (Ultimaker BV, Geldermalsen, The Netherlands).

### 2.2. Mechanical Testing

**Figure 2:**
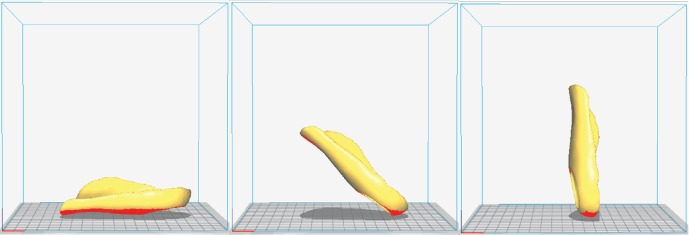
FOs printed for mechanical testing at different orientations. From left to right: 0°, 45°, and 90°.

**Figure 3:**
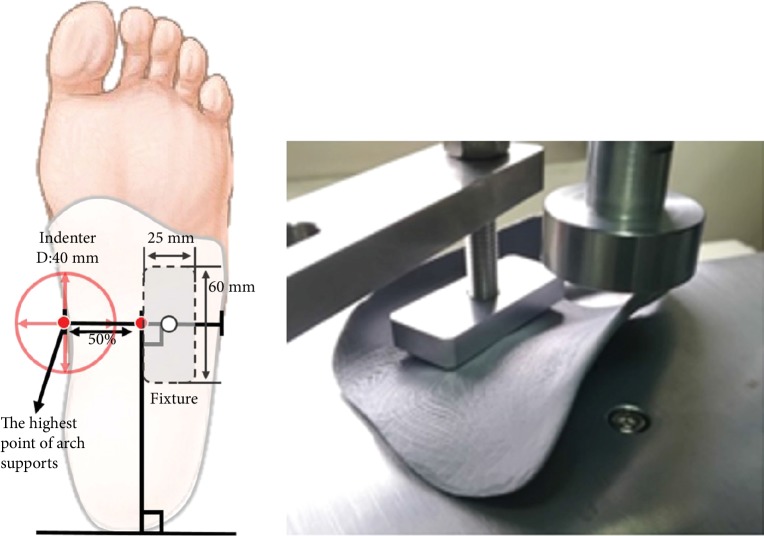
FO compressive testing procedure.

To obtain the mechanical characteristics of the FOs printed under three specific build orientations (elevation angles: 0°, 45°, and 90°; Figure 2), a total of 18 FO samples were fabricated and tested. Because no standard tests for FOs exist, we designed a procedure to test the stiffness of the FOs. During our mechanical testing, the FOs were fixed as illustrated in Figure 3. A rectangular fixture measuring 25 mm × 60 mm was placed on the lateral side of each FO. The FO was subjected to dynamic compression at a constant loading rate of 105 N/min by the testing machine (HT-2402, Hung Ta Instrument Co., Ltd., Taiwan) with a 40 mm diameter indenter. Six samples were tested for each build orientation. Displacement and reaction force data were collected.

### 2.3. Human Motion Analysis

Twelve individuals with flexible flatfoot (four male and eight female individuals; age: 25.92 ± 2.75 years; height: 1.66 ± 0.10 m; weight: 57.08 ± 10.03 kg) were enrolled in this study. The participants were selected according to the Foot Posture Index [12]. All participants had a total score greater than 6 and no current or past history of a diagnosable musculoskeletal, rheumatological, or orthopedic disorder of the lower extremity. Each participant's dominant leg was determined through a ball-kicking test. All testing procedures were approved by the Institutional Review Board of National Yang-Ming University, and participants' written informed consent was obtained before the experiments.

An eight-camera 3D Vicon (MX T20, Vicon Motion Systems Ltd., Oxford, UK) motion analysis system sampling at 100 Hz and an AMTI force plate (Advanced Mechanical Technology Inc., Watertown, USA) sampling at 1000 Hz were used to collect kinematic and kinetic data. Adapted from a previous study [13], reflective markers were placed bilaterally on the anterior and posterior superior iliac spine, greater trochanter, lateral and medial femoral epicondyles, head of the fibula, tibial tuberosity, and lateral and medial malleolus. To measure foot motion inside a standard shoe, a standard shoe was prepared with four additional cut-outs for placing markers at the heel, navicular tuberosity, and tuberosity of the second and fifth metatarsals.

Prior to data collection, the participants were given a 5-minute practice trial to familiarize themselves with the experimental surroundings. In addition, a static trial was captured to determine the joint center and the neutral joint orientations before each experimental condition. The participants were asked to perform five trials of level walking at a self-selected speed under two conditions, namely, walking in standard shoes (Shoe) and walking in standard shoes embedded with FOs (Shoe+FO). A 5-minute break was provided between conditions. The order of the experimental conditions was randomized across participants.

All kinematic and kinetic data of human motion were processed using Matlab (MathWorks Inc., Natick, MA, USA). The joint moments were calculated using the inverse dynamics method. The calculated joint moments were normalized by multiplying the body weight and leg length. The path of the center of pressure (COP) under the shoes was analyzed during the stance phase in walking, based on the COP position of the global coordinate system with respect to the locations of the heel and second metatarsal markers [14]. The maximum and minimum moment values at characteristic peaks during the stance phase were obtained from each participant's average curves across the five trials.

### 2.4. Statistical Analysis

A one-way analysis of variance (ANOVA) along with post hoc Tukey's test was performed to compare the maximum compressive load and stiffness of the 3D-printed FOs among the three different build orientations. In the human motion analysis, the peak hip, knee, and ankle joint moments and ground reaction forces (GRFs) were extracted. A paired-sample *t*-test was used to compare the peak joint moments and GRFs under the Shoe and Shoe+FO conditions. The statistical significance level was set as 0.05.

## 3. Results

### 3.1. Mechanical Testing

**Figure 4:**
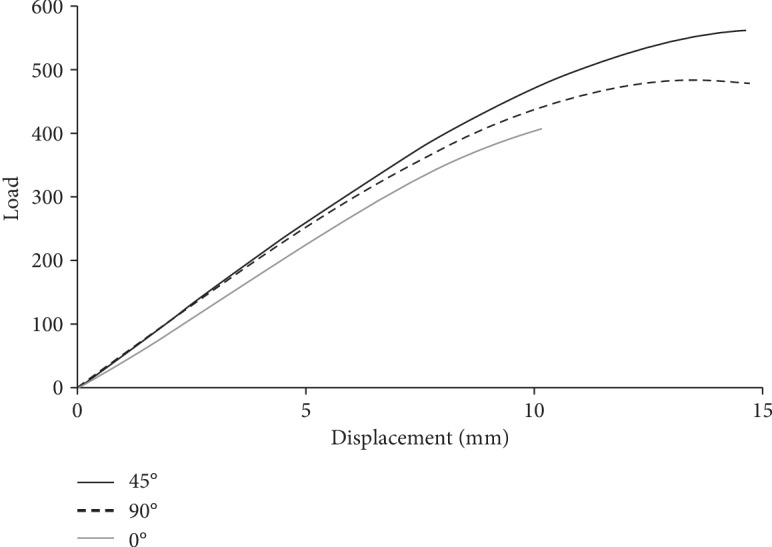
Average load-displacement curves for each build orientation.

The executed compressive tests revealed that the 45° and 90° build orientations engendered similar load and displacement behaviors in the FOs when the displacement was less than 5 mm (Figure 4). The ANOVA revealed differences between groups. The Tukey test demonstrated that the maximum load in the FOs fabricated using the 45° build orientation (563.58 ± 13.29 N) was significantly greater than those in the FOs fabricated using the 90° (494.46 ± 11.78 N) and 0° (401.42 ± 19.31 N) build orientations; the maximum load in the FOs fabricated using the 90° build orientation was significantly greater than those in FOs fabricated using the 0° one.

### 3.2. Human Motion Analysis

#### 3.2.1. GRF and COP

**Figure 5:**
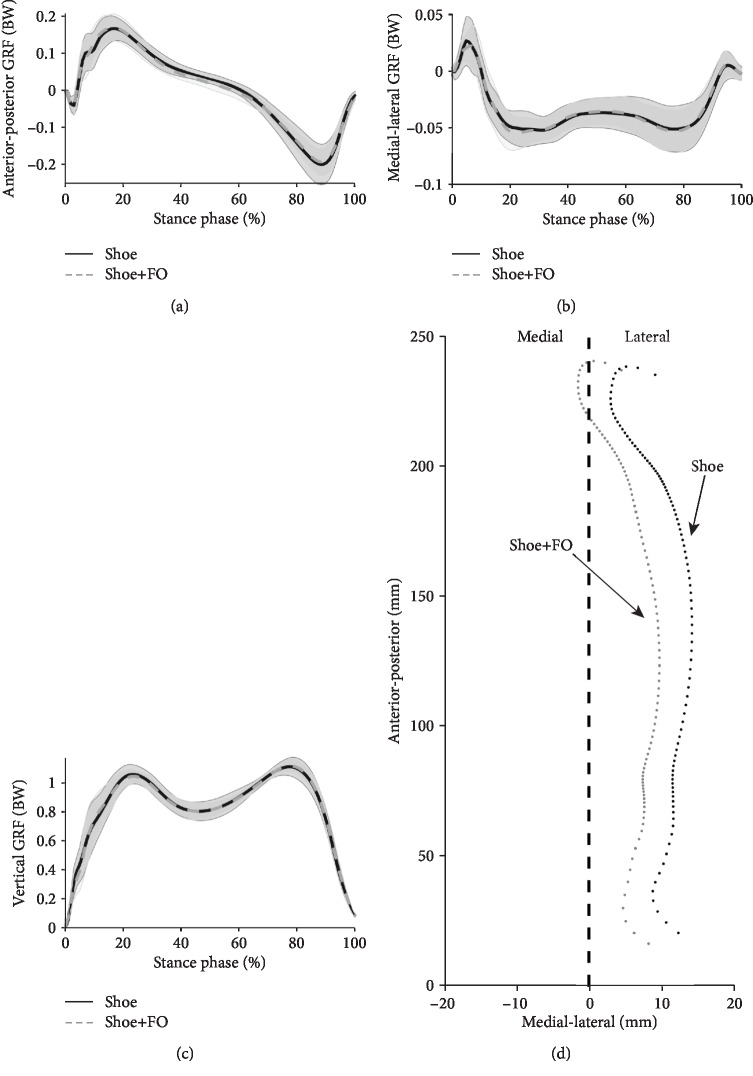
(a) Anterior-posterior, (b) media-lateral, and (c) vertical GRFs during walking. (d) Average path of the COP of shoes only (black dotted line) and of the shoes embedded with 3D-printed FOs (gray dotted line) with respect to the local coordinate system. The black dashed line represents the straight line between the heel and the second metatarsal markers. Abbreviations: BW: body weight.

No significant difference between the two experimental conditions (i.e., Shoe and Shoe+FO) was observed with respect to the maximum vertical, anterior-posterior, and medial-lateral GRFs (Table 1). The similar anterior-posterior (Figure 5(a)), medial-lateral (Figure 5(b)), and vertical (Figure 5(c)) GRF patterns were observed between the two different conditions. Notably, under the Shoe+FO condition, the 3D-printed FOs shifted the path of the COP medially by 4.3 mm on average compared with the Shoe condition (Figure 5(d)).

#### 3.2.2. Joint Moment

Table 2 reveals that the 3D-printed FOs significantly reduced the maximum ankle evertor moment by 35% on average. The peak external rotator moment observed for the shoe embedded with the 3D-printed FO was also reduced significantly by 16%. The maximum ankle plantar flexor moment under the Shoe+FO condition was significantly greater (+3%) than that under the Shoe condition. However, the two experimental conditions did not differ significantly in the maximum hip and knee joint moments for any of the three planes.

## 4. Discussion

The key findings of this study are that the use of 3D-printed FOs decreased the maximum ankle evertor and external rotator moments but increased the maximum ankle plantar flexor moment during walking. In addition, the use of 3D-printed FOs modified the COP path without altering the peak magnitude of the GRFs.

The primary treatment goal of FOs is to correct foot abnormalities. However, the function of 3D-printed FOs is not only to correct foot abnormalities but also to provide sufficient mechanical strength to bear the weight during walking. A previous study indicated that the outer shell of the specimen fabricated using a 0° build orientation was separated from the main body of the specimen during high-stress fatigue tests [10]. However, specimens fabricated using the 45° and 90° build orientations did not demonstrate the same gap problems. The finding that the 0° build orientation produced the weakest FOs might be explained by the wider gap between the outer shell and the main body during fabrication. To investigate the biomechanical effects of 3D-printed FOs in the lower extremities of humans, experimental 3D-printed FOs were fabricated by orientating the elevation angle at 45° because this orientation produced the strongest FOs.

During human walking, the plantar flexors stiffen the ankle, allowing the leg to rotate over a forefoot fulcrum [15]. Moreover, previous studies have demonstrated a considerable contribution of this plantar flexor push-off mechanism to the forward progression of the body [16, 17]. The ankle plantar flexor moment is primarily responsible for actual locomotion, whereas the moments in the frontal and transverse planes are primarily responsible for the dynamic stability of the lower extremities [6]. Compared with someone with a normal foot, an individual with flatfoot demonstrated greater muscle activation prior to push-off in the peroneus longus and the brevis, soleus, medial, and lateral gastrocnemius, reflecting a greater challenge in stabilizing the whole foot as the weight was transferred onto the toes [18]. Therefore, according to the results of this study, using 3D-printed FOs as interventions affected the ankle for actual locomotion by increasing the ankle plantar flexor moment, and it affected ankle moments highly associated with dynamic stability by decreasing the ankle evertor and external rotator moments.

The COP analysis results reveal that the path shifted toward the medial side under the Shoe+FO condition. A possible explanation for this finding is that the contour of the corresponding 3D-printed FO was based on the 3D scanning model obtained in the subtalar neutral position; therefore, the FO maintained the foot posture in the neutral position as much as possible during the stance phase. The structural materials of both soft and rigid FOs must be able to retain the foot shape during walking [19]. The different degrees of hardness of EVA or PP are most often used to fabricate conventional semirigid FOs. Although the Young modulus of PLA (3.5 GPa) is higher than PP (1.5 to 2 GPa), the flexibility of 3D-printed PLA FOs is similar to PP FOs [20]. Therefore, the 3D-printed FOs for this study is semirigid. The current study demonstrated that the 3D-printed FOs fabricated using PLA could retain the foot shape. This might lead to the COP path shifting toward the midline.

The changes in the COP path in the anterior-posterior and medial-lateral directions could affect the ankle moments in the sagittal and frontal plane, respectively [21]. A similar GRF pattern was observed between the two different conditions. We believe that the changes in ankle moments were associated with the changes in the COP path when the 3D-printed FOs were worn. Moreover, no correlation was observed between changes in the COP path and changes in knee joint moments [6]. In the present study, the 3D-printed FOs did not change the peak knee joint moments in individuals with flexible flatfoot. The 3D-printed FOs may not have changed the knee axial alignment either. A possible explanation is that the individuals with flatfoot who were enrolled for this study may not have severe problems of malalignment of the lower extremities. Therefore, the 3D-printed FOs did not alter the knee and hip joint moments.

The fabrication costs for a pair of conventional custom-made FO ranged from 194 to 485 USD in Taiwan. The cost of a 3D-printed product based on the weight of the device and the 3D-printed FOs are around 90 gram and material costs only about 2 USD. The 3D scanner and desktop FDM 3D printer cost 710 USD and 1450 USD, respectively. In addition, a number of companies have added 3D print-on-demand services to their offering. The 3D printing services cost about 73 USD to fabricate FOs in Taiwan. According to above, the 3D-printed FOs cost lower than the traditional fabrication.

Several limitations of this study must be considered. First, this study focused on the immediate effects of 3D-printed FOs in individuals with flexible flatfoot and did not identify long-term responses. Second, we used kung-fu shoes as the standard shoes in this study. Kung-fu shoes are cheaply produced with minimal lining. They have a low-sided cloth upper and a flat hard plastic sole. However, the effects of 3D-printed FOs in different types of shoes, such as running shoes, remain unclear.

## 5. Conclusion

This study demonstrated that 3D-printed FOs with different orientations produce different mechanical properties. The 45° build orientation produced the strongest FOs. In addition, the 3D-printed FOs engendered a decrease in ankle evertor and external rotator moments by changing the COP path medially, but they induced an increase in the ankle plantar flexor moment. We can conclude that the low-cost 3D printing technology has the capability of fabricating custom FOs with sufficient support to correct foot abnormalities. We provide evidence that such FOs engender biomechanical changes and positively influence individuals with flexible flatfoot.

## Figures and Tables

**Table 1:** Peak GRFs during walking under two conditions: wearing standard shoes (Shoe) and wearing shoes embedded with 3D-printed FOs (Shoe+FO).

Peak GRF (BW)	Shoe (mean ± SD)	Shoe+FO (mean ± SD)	*p* value	Effect size
Anterior	−0.20 ± 0.05	−0.20 ± 0.04	0.41	0.14
Posterior	0.17 ± 0.04	0.18 ± 0.04	0.84	0.04
Medial	−0.06 ± 0.02	−0.07 ± 0.01	0.39	0.21
Lateral	0.03 ± 0.02	0.03 ± 0.02	0.57	0.12
Vertical	1.12 ± 0.06	1.12 ± 0.05	0.66	0.06

BW: body weight.

**Table 2:** Comparison of the maximum joint moments at the ankle, knee, and hip during walking between two conditions: wearing standard shoes (Shoe) and wearing shoes embedded with 3D-printed FOs (Shoe+FO).

Joint	Moment (Nm/BW/LL)	Shoe (mean ± SD)	Shoe+FO (mean ± SD)	*p* value	Effect size
Ankle	Invertor	0.012 ± 0.005	0.011 ± 0.005	0.14	0.29
	Evertor	−0.007 ± 0.005	−0.005 ± 0.004	0.04^∗^	0.53
	Internal rotator	0.005 ± 0.003	0.005 ± 0.004	0.51	0.14
	External rotator	−0.061 ± 0.011	−0.051 ± 0.015	0.03^∗^	0.72
	Dorsiflexor	0.020 ± 0.007	0.020 ± 0.009	0.93	0.01
	Plantarflexor	−0.161 ± 0.013	−0.167 ± 0.013	0.04^∗^	0.41

Knee	Adductor	0.014 ± 0.006	0.018 ± 0.009	0.12	0.57
	Abductor	−0.041 ± 0.018	−0.044 ± 0.018	0.17	0.17
	Internal rotator	0.003 ± 0.003	0.004 ± 0.003	0.18	0.21
	External rotator	−0.017 ± 0.006	−0.018 ± 0.006	0.59	0.08
	Extensor	0.067 ± 0.017	0.068 ± 0.022	0.86	0.04
	Flexor	−0.031 ± 0.006	−0.032 ± 0.006	0.33	0.20

Hip	Adductor	0.011 ± 0.010	0.014 ± 0.014	0.11	0.29
	Abductor	−0.102 ± 0.016	−0.105 ± 0.020	0.39	0.16
	Internal rotator	0.018 ± 0.006	0.019 ± 0.009	0.54	0.08
	External rotator	−0.019 ± 0.014	−0.018 ± 0.014	0.65	0.10
	Flexor	0.159 ± 0.026	0.170 ± 0.049	0.24	0.27
	Extensor	−0.096 ± 0.010	−0.099 ± 0.012	0.34	0.33

^∗^Significantly different between the two conditions. BW: body weight, LL: leg length.

## Data Availability

The data within this study are archived in the Computational Biomechanics Laboratory of the Department of Physical Therapy and Assistive Technology at National Yang-Ming University. The corresponding author can be contacted for inquiries.
